# MicroRNA-16 suppresses metastasis in an orthotopic, but not autochthonous, mouse model of soft tissue sarcoma

**DOI:** 10.1242/dmm.017897

**Published:** 2015-08-01

**Authors:** Mohit Sachdeva, Melody J. Whitley, Jeffrey K. Mito, Yan Ma, Dina C. Lev, Diana M. Cardona, David G. Kirsch

**Affiliations:** 1 Department of Radiation Oncology, Duke University Medical Center, Durham, NC NC27708, USA; 2 Department of Pharmacology & Cancer Biology, Duke University Medical Center, Durham, NC 27708, USA; 3 Department of Cancer Biology, University of Texas, MD Anderson Cancer Center, Houston, TX 77054, USA; 4 Department of Pathology, Duke University Medical Center, Durham, NC 27708, USA

**Keywords:** Genetic engineering, Mouse models, Metastasis, MicroRNA, Soft tissue sarcoma

## Abstract

MicroRNAs (miRNAs) can regulate tumor cell invasion and metastasis in a tumor-specific manner. We recently demonstrated that global downregulation of miRNAs after deleting dicer can promote development of distant metastases in a mouse model of primary soft tissue sarcoma (STS). In this study, we identified miRNAs that are differentially downregulated in metastatic STS in both human and mouse, and investigated the role of these miRNAs in metastasis. miRNA- TaqMan PCR arrays showed a global downregulation of miRNAs in metastatic human sarcomas. Similar analysis in mouse metastatic sarcomas revealed overlap for several downregulated miRNAs including miR-16, miR-103, miR-146a, miR-223, miR-342 and miR-511. Restoration of these downregulated miRNAs in mouse primary sarcoma cell lines showed that miR-16, but not other downregulated miRNAs, was able to significantly suppress both migration and invasion *in vitro*, without altering cell proliferation. In addition, orthotopic transplantation of a sarcoma cell line stably expressing miR-16 into the muscle of immunocompromised mice revealed that restoration of miR-16 can significantly decrease lung metastasis *in vivo*. However, no change in the rate of lung metastasis was observed when miR-16 was deleted in mouse primary sarcomas at sarcoma initiation. Taken together, these results indicate that miR-16 can have metastasis-suppressing properties both *in vitro* and *in vivo*. However, the loss-of-function experiments in autochthonous tumors indicate that loss of miR-16 is not sufficient to promote metastasis *in vivo*.

## Abstract (precis)

**Summary:** This work underscores the importance of studying cancer metastasis using *in vivo* model systems in addition to *in vitro* and transplant model systems.

## INTRODUCTION

Despite important advances in cancer research and cancer therapies, the primary cause of mortality for cancer patients is still metastasis (Lyman and Djulbegovic, 2005). Metastases arise in a multi-step process. First, tumor cells locally invade into the stroma, followed by intravasation of the primary tumor into the circulation, then extravasation into the secondary site, and finally, colonization of the cells to form secondary tumors (Nguyen et al., 2009). Fatal lung metastases occur in approximately one-third of patients with soft tissue sarcomas (STS), which are a diverse group of malignant tumors that arise from mesenchymal tissues (Borden et al., 2003). Currently, there are no molecular markers to predict which sarcoma patients will develop metastasis. Therefore, a deeper understanding of sarcoma metastasis may help to identify patients at high risk of developing metastasis. Accumulating evidence suggests that tumor cell invasion and metastasis is regulated by microRNAs (miRNAs) (White et al., 2011). For example, we recently showed that a single miRNA (miR-182) modulated sarcoma metastasis *in vivo* (Sachdeva et al., 2014). miRNAs are small non-coding RNA molecules composed of 20–22 nucleotides. Genes encoding miRNA are generally transcribed by RNA polymerase (Pol II) to primary transcripts (pri-miRNAs), which are cleaved to produce stem–loop structured precursors (pre-miRNAs) that are subsequently processed into mature miRNAs. One strand of the miRNA duplex is then loaded into the RNA-induced silencing complex (RISC), which mediates gene suppression through mRNA degradation or translational repression with the miRNA binding to the target mRNA (Ambros, 2001; Bartel, 2004). Deregulation of miRNA expression is often associated with a variety of disorders, including human malignancy (Lu et al., 2005).

**Fig. 1.**
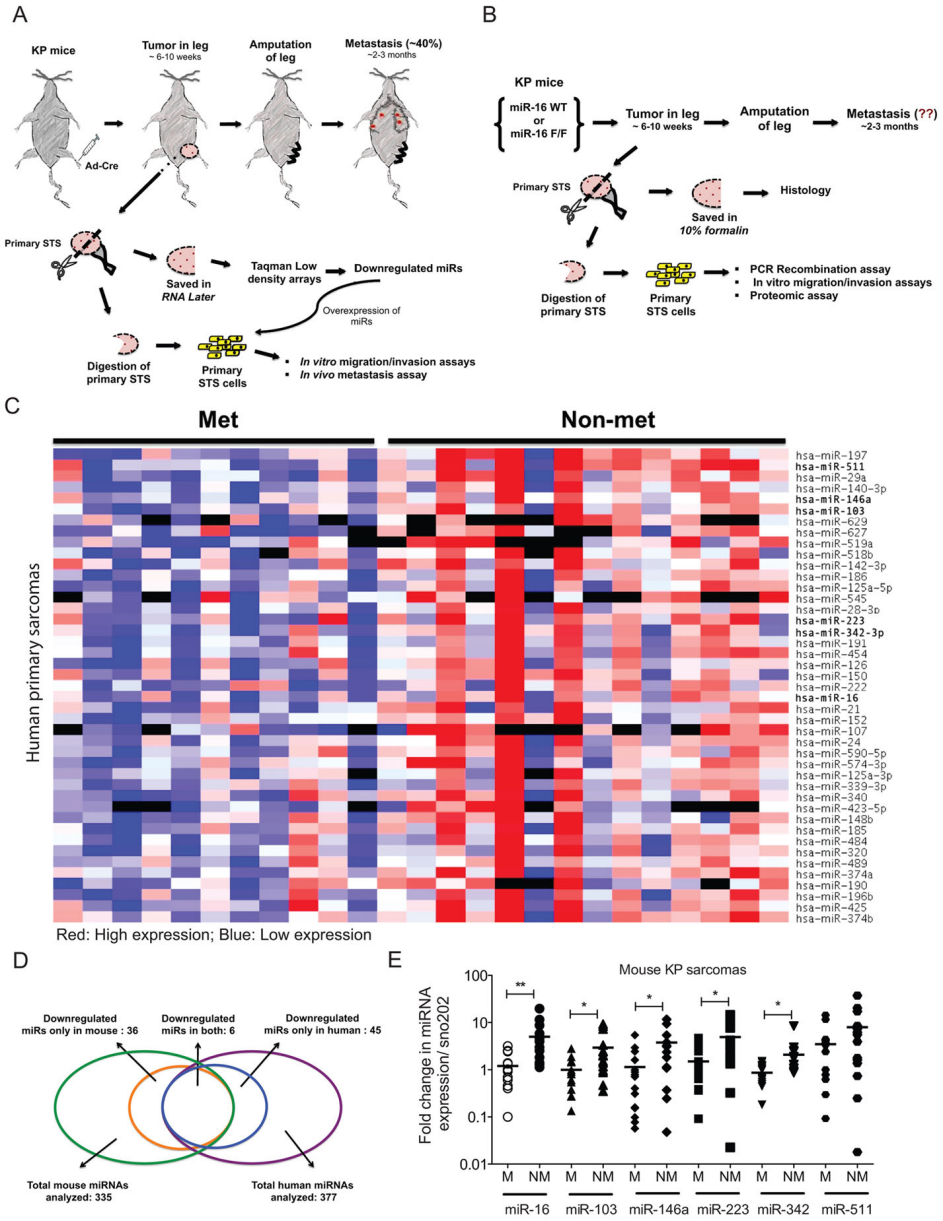
**Global downregulation of miRNAs in both human and mouse metastatic sarcomas.** Schematics showing study design for modeling sarcoma metastasis (A) and studying the role of downregulated miRNAs in metastasis (B). (C) Heat map showing differential expression of miRNAs between non-metastatic (*n*=14) versus metastatic (*n*=11) primary human sarcomas using TaqMan PCR arrays with 377 miRNAs (blue: low expression; red: high expression). 45 miRNAs were downregulated. (D) miRNA expression was analyzed in 335 mouse sarcomas (green circle) and 337 human sarcomas (purple circle). 36 miRNAs were downregulated in metastatic mouse sarcomas (orange circle) and 45 miRNAs were found to be downregulated in metastatic human sarcomas (blue circle). This Venn diagram shows the overlap of those two groups, with 6 miRNAs downregulated in both mouse and human metastatic tumors. (E) The relative expression of these miRNAs in metastatic (M) and non-metastatic (NM) primary mouse sarcomas is shown by qPCR. Two-tailed Student *t*-test was used to evaluate the statistical significance of individual miRNAs between metastatic (*n*=25) and non-metastatic (*n*=39) sarcomas. *P<0.05, **P<0.001. Note that names of miRNAs downregulated in both mouse and human metastatic tumors are in bold in C.

To study sarcoma metastasis, our lab has developed a genetically engineered mouse model of STS with conditional mutations in *Kras* and *p53* (*LSL-Kras^G12D^; p53^flox/flox^*; known as ‘KP’ mice) (Kirsch et al., 2007). When these oncogenic mutations are activated in KP mice by injection of adenovirus expressing Cre recombinase (Ad-Cre), the KP mice develop high-grade primary STS at the site of injection in 2-4 months (Fig. 1A,B). Because sarcomas develop in a spatially and temporally restricted manner, the primary tumor can be surgically removed, and the mice can be subsequently monitored for the development of secondary lung tumors as a measure of the metastatic potential of the primary tumor. We have observed that approximately 40% of KP sarcomas spread to the lungs (Mito et al., 2009). In the current study, we identified miRNAs that are downregulated in metastatic sarcomas in mice and humans, and investigated the role of these miRNAs in regulating sarcoma metastasis.

## RESULTS

### Global downregulation of miRNAs in both human and mouse metastatic sarcomas

We previously showed that decreasing miRNA biogenesis promotes metastasis of primary STS in KP mice (Mito et al., 2013). Using unbiased miRNA TaqMan PCR arrays, we profiled resected primary sarcomas from KP mice. We observed that the majority of miRNAs were downregulated in tumors that metastasized to the lungs (Sachdeva et al., 2014). To extend this finding to human sarcomas, we profiled miRNAs from human primary undifferentiated pleomorphic sarcoma (UPS) with known metastatic outcome. Similar to the mouse sarcomas, we observed a global downregulation of miRNAs in sarcomas that metastasized compared with those that did not spread (Fig. 1C and supplementary material Table S1). By comparing the downregulated miRNAs in metastatic sarcomas from human and mouse, we found six miRNAs common to both: miR-16, miR-103, miR-146a, miR-223, miR-342 and miR-511 (Fig. 1D,E). These six miRNAs were therefore selected for further study. Notably, some of these miRNAs, such as miR-146a and miR-223, have also been implicated in modulating metastasis in other tumor types (Hurst et al., 2009; Li et al., 2011).

**TRANSLATIONAL IMPACT**

**Background**

The primary cause of mortality among patients with cancer is metastasis, the formation of secondary tumors distant from the original tumor site. During metastasis, primary tumor cells invade locally into the stroma (the connective tissue that surrounds the organs of the body) before entering the circulation and travelling round the body in the blood. Eventually, the tumor cells exit the circulation and form secondary tumors or metastases. Approximately one-third of patients with soft tissue sarcomas (STS; malignant tumors that arise from mesenchymal tissues such as muscle), for example, develop fatal lung metastasis. Unfortunately, there are currently no molecular markers that can be used in the clinic to predict which patients with STS are at risk for developing distant metastases.

**Results**

To discover biomarkers that correlate with the development of metastasis, the authors analyze the expression of microRNAs (miRNAs; small non-coding RNA molecules that can regulate tumor cell invasion and metastasis) in human and mouse STS with known metastatic outcomes. They report that miR-16 is downregulated in both human and mouse metastatic STS. Restoration of miR-16 expression in mouse primary sarcoma cell lines significantly suppressed both migration and invasion *in vitro* and metastasis to the lung of sarcoma cells transplanted into the muscle of immunocompromised mice (an orthotopic mouse model of STS). Notably, however, there was no change in the rate of lung metastasis when miR-16 was deleted in autochthonous tumors (a tumor that forms where it is found rather than being transplanted from elsewhere) in a mouse model of primary STS previously developed by the authors in which sarcomas develop in a spatially and temporally restricted manner and can be surgically resected so that the true metastatic potential of the primary tumor can be determined.

**Implications and future directions**

Metastasis is a complex process that is not very well understood. Many studies have used immunocompromised mouse models to study metastasis. However, in such models, it is difficult to study the interaction between the tumor cells, the tumor microenvironment and the host's immune response. The discordant results reported here between the effects of miR-16 overexpression in an orthotopic transplant model in immunocompromised mice and miR-16 deletion in a primary tumor model in immunocompetent mice demonstrate the importance of utilizing complementary gain-of-function and loss-of-function approaches and primary tumor model systems for the study of metastasis.

### miR-16, but not other miRNAs, suppresses both migration and invasion *in vitro*

**Fig. 2.**
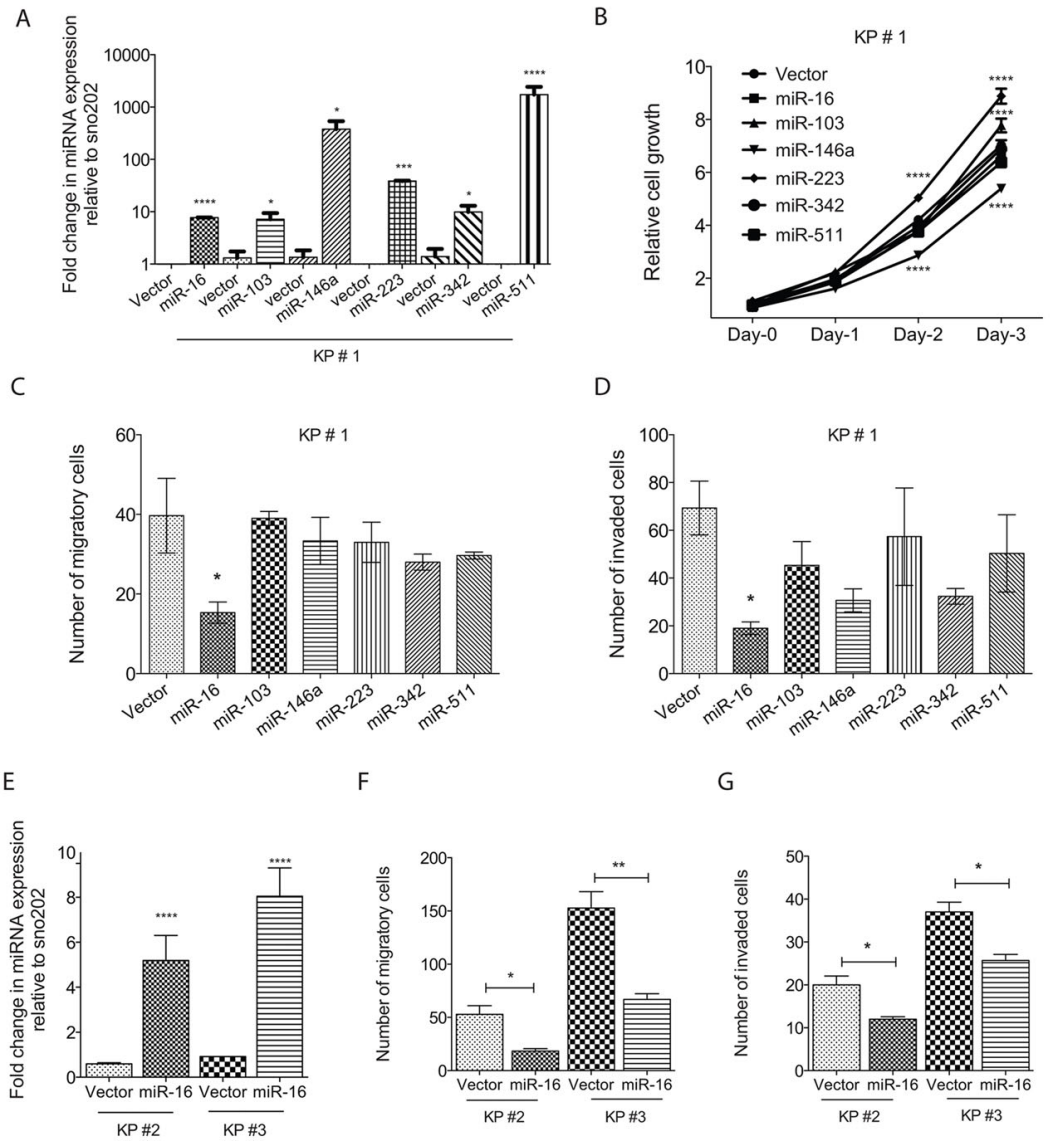
**Effect of miRNAs on migration and invasion of primary sarcoma cell lines.** (A) Real-time PCR demonstrating ectopic expression of stably transduced miRNAs relative to reference sno202. For each miRNA, three biological replicates were performed and the data are presented as the means±s.d. (B) Cell growth assay showing that ectopic expression of miRNAs does not affect cell proliferation *in vitro* with the exception of miR-223, which enhances the growth at days 2 and 3, and miR-146a, which suppresses cell growth at days 2 and 3. (C,D) Quantification of Matrigel assay demonstrating that miR-16, but not any of the other miRNAs, suppresses both migration and invasion, respectively. (E) Real-time PCR demonstrating ectopic expression of miR-16 relative to reference sno202. (F,G) miR-16 overexpression suppresses migration and invasion of sarcoma cell lines KP2 and KP3. Values in C-G are means±s.e. of three independent experiments. One-way ANOVA is used for statistical analysis in A-D and two-tailed Student's *t*-test in E-G. *P<0.05, **P<0.001, ***P<0.0005, ****P<0.0001.

Global downregulation of miRNAs has been shown to correlate with both high-grade tumors and poor patient survival (Lu et al., 2005; Martello et al., 2010). To test whether the six candidate downregulated miRNAs can alter *in vitro* phenotypes of metastasis, we stably re-expressed these six miRNAs in a primary sarcoma cell line derived from KP mice and confirmed overexpression of each miRNA using TaqMan real-time PCR (Fig. 2A). Previous studies have shown that miR-16 can suppress cell cycle progression by targeting multiple G1 cyclins (Bandi et al., 2009) and that miR-223 can indirectly regulate cyclin E (Xu et al., 2010). Therefore, we tested whether any of these miRNAs altered STS cell proliferation. Overexpression of these miRNAs had no significant effect on cell proliferation, with the exception of miR-223, which increased cell proliferation, and miR-146a, which decreased cell proliferation (Fig. 2B). We then determined whether overexpression of any of these miRNAs impacts *in vitro* cell migration and invasion by culturing cells in Matrigel chambers. We scored the number of cells that migrated towards the serum-containing cell culture medium or invaded through the Matrigel, which is a membrane composed of matrix proteins. Unlike the other miRNAs tested, overexpression of only miR-16 significantly decreased both migration and invasion of KP cells (Fig. 2C and D, respectively).

Cells overexpressing miR-146a and miR-342 showed a decrease only in the invasion assay, not in migration. However the decreased invasion was not statistically significant. Interestingly, despite miR-103 being the most downregulated miRNA in both human and mouse metastatic sarcomas, restoration of miR-103 had no effect on either migration or invasion. To further characterize the role of miR-16 on migration and invasion of KP cells and to exclude the possibility that this miR-16 phenotype was restricted to KP cell line 1 (KP1), we stably expressed miR-16 in two additional KP cell lines (KP2 and KP3; Fig. 1E). Overexpression of miR-16 significantly decreased both migration and invasion in both of these cell lines *in vitro*, without altering their cell proliferation (Fig. 1F,G and data not shown). These results demonstrate that miR-16 can suppress migration and invasion *in vitro* in multiple KP sarcoma cell lines without altering cell proliferation. Therefore, we next investigated whether miR-16 modulates sarcoma metastasis *in vivo*.

### miR-16 overexpression suppresses lung metastasis in a nude mouse transplant model *in vivo*

To determine whether miR-16 can suppress metastasis *in vivo*, we transplanted sarcoma cells into the muscle of nude mice so that the tumor cells would grow at an orthotopic site. In this model, primary sarcoma cells with or without miR-16 overexpression are injected into the muscle of immunocompromised mice and after tumors developed they were resected and the mice were followed for the development of lung metastasis.

**Fig. 3.**
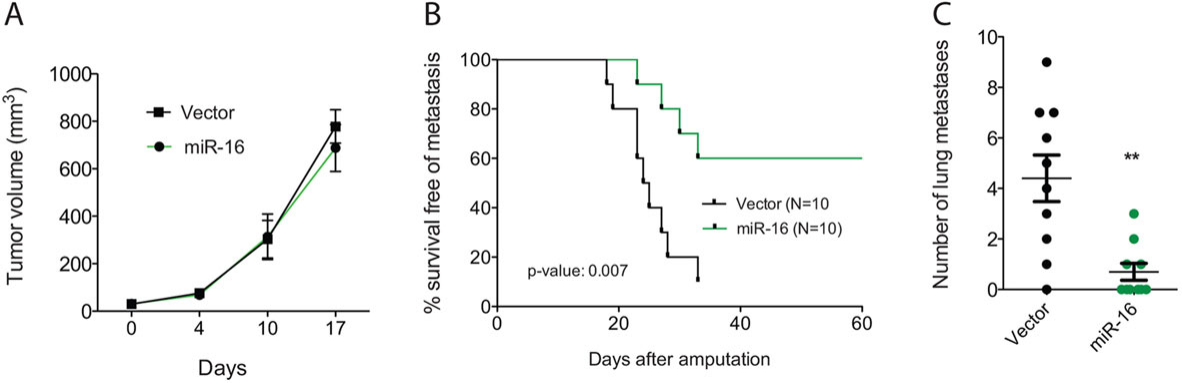
**miR-16 overexpression suppresses lung metastasis in a nude mouse transplant model *in vivo*.** (A) Tumor growth curves of orthotopic sarcomas expressing vector control or miR-16 shows that ectopic expression of miR-16 has no effect on *in vivo* growth of sarcomas. (B) After surgical resection of orthotopic tumors in the lower extremity, mice were followed for the development of lung metastases (i.e. secondary tumors). Mice with tumors overexpressing miR-16 were more likely to survive without metastasis to the lung. Data are shown in the Kaplan–Meier plot as percentage survival free of metastasis. (C) Lungs from mice in B were analyzed and the number of lung metastases identified in each mouse is shown. The lungs from mice in which orthotopic tumors overexpressed miR-16 had a decreased number of lung metastases. Mantel–Cox log-rank test is used in B and two-tailed Student's *t*-test is used for statistical analysis in C. **P<0.01.

For injection, we utilized an STS cell line derived from a primary tumor that had a high base rate of metastasis (KP1), allowing us to test whether miR-16 overexpression can suppress metastasis. Nude mice lack a functional immune system so the tumor cells were able to graft easily. As shown in Fig. 3A, there was no change in the growth of primary tumors between vector control and miR-16-infected cells, but a significant difference was observed in the rate and number of lung metastases (Fig. 3B,C). For example, 9/10 mice developed lung metastasis in the vector control group, but only 4/10 mice developed lung metastasis from the sarcoma cells overexpressing miR-16. The average number of lung metastases per mouse was 4.5 with vector-infected cells and 1 with miR-16-infected cells (Fig. 3C). These results are consistent with the *in vitro* experiments in which overexpression of miR-16 decreased metastatic phenotypes of invasion and migration (Fig. 2). Taken together, these *in vitro* and *in vivo* results suggest that miR-16 can act as a metastasis suppressor in sarcoma.

### Deletion of miR-16 does not increase metastasis in a mouse model of primary STS

**Fig. 4.**
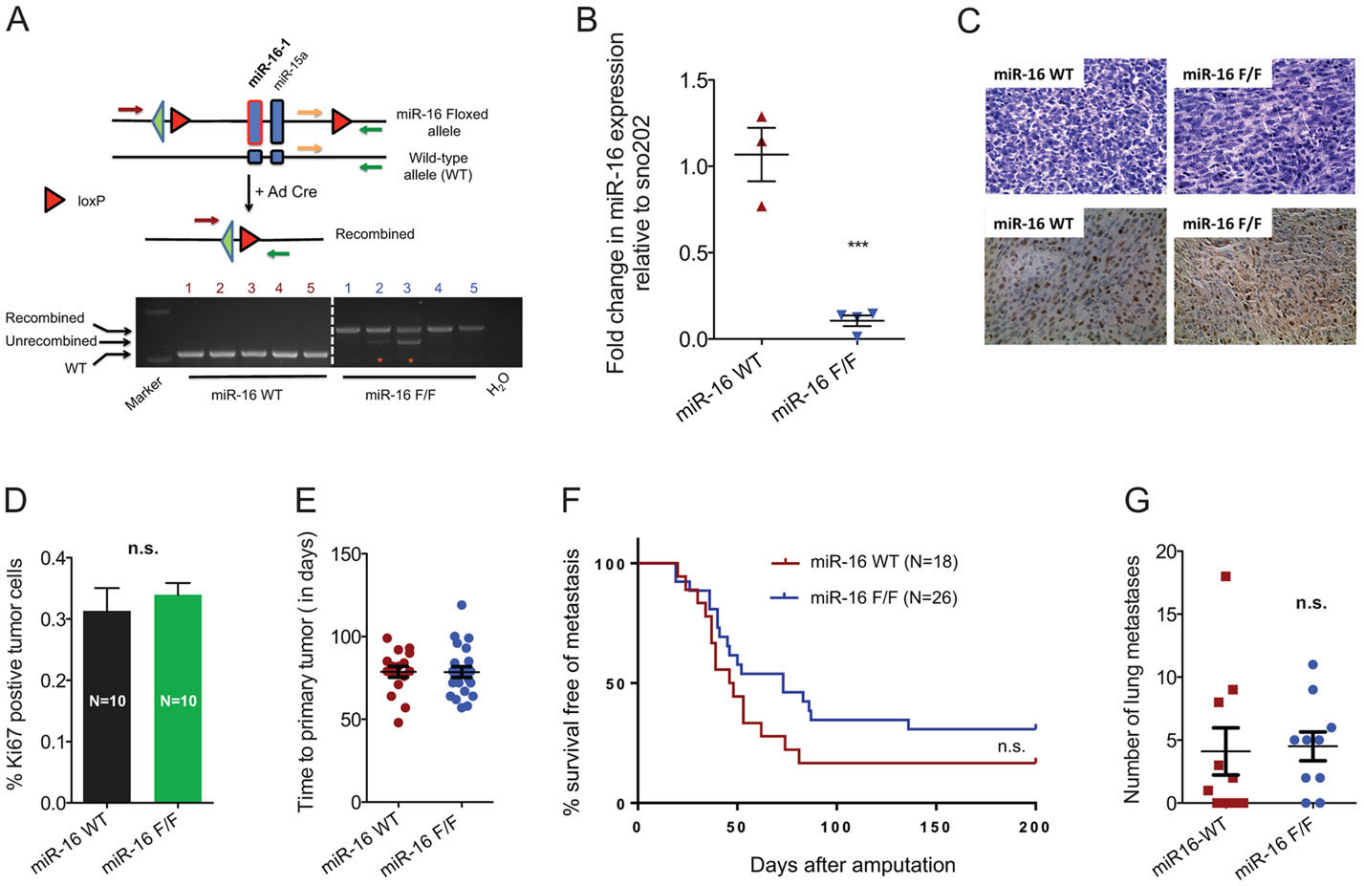
**Deletion of miR-16 fails to promote lung metastasis in a mouse model of primary sarcoma.** (A) Schematic showing deletion of miR-16 by an adenovirus expressing Cre recombinase (Ad-Cre) (top panel). PCR showing Cre-mediated excision of miR-16 with the recombined allele from primary sarcomas (bottom panel). Red asterisks denote samples with either partial recombination of the miR-16 flox allele or stromal contamination in the primary sarcoma cell line. The cell lines derived from independent tumors are numbered 1-5 (red, miR-16 WT; blue, miR-16 F/F). (B) TaqMan PCR for miR-16 relative to reference sno202 demonstrates decreased miR-16 expression in sarcomas from KP miR-16 F/F mice compared with control KP miR-16 WT mice. (C) Sarcomas with or without miR-16 deletion appear similar by histology after staining with hematoxylin and eosin (top panels) or after Ki67 immunohistochemistry (bottom panels). (D) Quantification of Ki67 staining (*n*=10 per genotype for the number of tumors in which a section of tumor was analyzed. (E) Time to tumor development after Ad-Cre injection. There is no impact of miR-16 deletion on time to sarcoma development (*N*=18 control KP miR-16 WT mice and *N*=26 KP-miR-16 F/F mice). (F) After surgical resection of primary tumors in the lower extremity, mice were followed for the development of lung metastases (i.e. secondary tumors). Mice with tumors with deletion of miR-16 have similar survival without metastasis. Data are shown in the Kaplan–Maier plot as percentage survival free of metastasis. (G) miR-16 did not decrease the number of lung metastases to the lungs. Mantel–Cox log-rank test is used in F and two-tailed Student's *t*-test is used for statistical analysis in B,D,E,G. ***P<0.005; n.s., not significant.

To further test the functional significance of miR-16 as a metastasis suppressor gene, we used a loss-of-function approach in primary sarcomas. We crossed KP mice to mice in which miR-16 was flanked by *loxP* sites (i.e. miR-16^flox/flox^ mice) so that Cre recombinase can delete miR-16 (Fig. 4A). We generated primary sarcomas by injecting an adenovirus expressing Cre recombinase (Ad-Cre) into KP mice (miR-16 WT) and into KP miR-16^flox/flox^ mice to generate sarcomas with deletion of miR-16 (miR-16 F/F) (Fig. 4A). After Ad-Cre injection, KP mice with miR-16 deletion developed primary sarcomas with similar kinetics to the mice with wild-type miR-16. PCR analysis on genomic DNA from primary cell lines generated from sarcomas with or without miR-16 deletion demonstrated efficient recombination of the miR-16 allele in KP sarcomas (Fig. 4A). In addition, qRT-PCR confirmed that miR-16 expression is lost at the transcript level in the primary cell lines (Fig. 4B). Because miR-16 has been shown to suppress cell proliferation in various tissues, we next determined whether deletion of miR-16 had any effect on proliferation of sarcoma cells. Of note, immunostaining of sarcomas with an antibody directed against Ki-67, which is a marker of cellular proliferation because it is expressed during interphase (G1, S, G2) and mitosis (M), but not in resting G0 cells (Scholzen and Gerdes, 2000), showed no difference between the two groups suggesting that miR-16 deletion had no significant impact on proliferation of sarcoma cells *in vivo* (Fig. 4C,D). Confirming that miR-16 is efficiently deleted in primary sarcomas and with no effect on sarcoma proliferation, we expanded a cohort of KP miR-16^flox/flox^ and KP miR-16^wt/wt^ mice to generate primary sarcomas to study metastasis. We did not observe a significant change in tumor onset or tumor growth kinetics after deletion of miR-16 in KP tumors (Fig. 4E). Sarcomas were surgically removed by amputation of the tumor-bearing limb. The amputated mice were then observed for a minimum of 6 months for the development of distant lung metastases. To our surprise, and in contrast to our findings in the orthotopic metastasis assay, we did not observe any change in the rate of lung metastases in the sarcomas with or without deletion of miR-16 (Fig. 4F). Moreover, no difference in the average number of lung metastases per mouse was observed between the two phenotypes (Fig. 4G). This finding suggests that decreasing miR-16 expression is not sufficient to promote lung metastasis in primary sarcomas in KP mice.

## DISCUSSION

Deregulation of microRNA expression has been reported in a variety of human diseases, especially cancer (Esquela-Kerscher and Slack, 2006; Iorio and Croce, 2012). For example, several studies have observed a global downregulation of miRNAs in a variety of tumors compared with normal tissues, suggesting a possible role of miRNAs in tumor initiation and progression (Lu et al., 2005). Additionally, haploinsufficiency in Dicer, a protein responsible for miRNA biogenesis, can promote tumorigenesis (Kumar et al., 2009) and suppression of *Dicer* expression by miR-103/107 attenuates miRNA biogenesis and promotes metastasis (Martello et al., 2010). We have shown that conditional deletion of *Dicer*, in the appropriate genetic context, promotes sarcoma progression *in vivo* in mice (Mito et al., 2013). In this study, we report that miRNAs are globally downregulated in both human and mouse primary sarcomas that give rise to distant metastases. We further demonstrate that overexpression of miR-16 decreases metastasis in an orthotopic mouse model in immunocompromised mice, but deletion of miR-16 in primary sarcomas fails to increase metastasis.

In the current study, using TaqMan PCR arrays, we observed a global downregulation of miRNAs in both human and mouse metastatic sarcomas. This finding is consistent with the observation that miRNAs are downregulated in high-grade tumors (Lu et al., 2005). However, it remained unclear which of the downregulated miRNAs contributed to sarcoma metastasis. Using a series of *in vitro* and *in vivo* experiments, we demonstrate that restoration of downregulated miRNAs can impact invasion and migration of cells *in vitro*. For example, stable overexpression of miR-16 suppresses both migration and invasion of cells in a Matrigel assay without affecting proliferation of the cells. In addition, in an orthotopic amputation model, miR-16 overexpression in a sarcoma cell line (KP1) suppresses both the rate and number of lung metastases. This finding suggests that restoration of miR-16 may downregulate gene(s) responsible for cellular migration and invasion.

Accumulating evidence suggests that miRNAs can regulate cell invasion and metastasis. For example, we recently showed that miR-182 drives metastasis of primary sarcomas *in vivo* (Sachdeva et al., 2014). Using novel genetically engineered mice to either delete or overexpress miR-182 in primary sarcomas *in vivo*, we showed that deletion of miR-182 in primary sarcomas significantly decreased the rate of lung metastasis after amputation of the tumor-bearing limb, whereas overexpression of miR-182 significantly increased the rate of lung metastasis (Sachdeva et al., 2014). One strength in studying the impact of miRNA in metastasis using primary tumors in genetically engineered mice is that the tumors develop within a native microenvironment in an immunocompetent host. Therefore, the interaction among the tumor cells, the tumor microenvironment and the host's immune response can all be studied in autochthonous tumors in mice. For example, miR-29, a miRNA regulated by GATA3, inhibits metastasis by targeting a network of pro-metastatic regulators involved in angiogenesis, collagen remodeling and proteolysis, suggesting that a miRNA can function by altering the tumor microenvironment (Chou et al., 2013; Melo and Kalluri, 2013). Therefore, to better define the role of miR-16 in metastasis, we utilized a primary model of STS in immunocompetent mice. Using miR-16^flox/flox^ mice, we were able to delete two alleles of miR-16 during sarcomagenesis and then follow the mice for the development of lung metastases. To our surprise, we did not observe any change in the rate of lung metastasis after deleting miR-16. There are several possible explanations for this result. First, the primary tumor model system examined miR-16 deletion. This loss-of-function experiment suggests that deletion of miR-16 is not sufficient to promote metastasis. However, it does not rule out the possibility that, in concert with downregulation of other miRNAs, loss of miR-16 could contribute to metastasis. This might explain the apparent discordance with the orthotopic transplant experiments in which miR-16 overexpression decreased metastasis, indicating that high levels of miR-16 suppress metastasis. However, these orthotopic transplant experiments do not address whether decreased miR-16 expression is sufficient to promote metastasis. Alternatively, the results from the primary tumor experiment might simply reflect the fact that there was an unexpectedly high rate of lung metastasis in the control KP mice, which could be a consequence of the mixed genetic background of the miR-16^flox/flox^ mice. Therefore, deletion of miR-16 in this experimental system may have been unable to further promote metastasis. Finally, it is possible that the discordant results between the two *in vivo* approaches reflect differences between a primary tumor model system with an intact immune system and a transplant model system in immunodeficient mice.

To search for potential targets of miR-16 that can regulate metastasis, we performed a liquid chromatography/mass spectrometry (LC/MS) proteomic screen of cell lines derived from primary sarcomas with (*n*=3) or without (*n*=3) miR-16 deletion, as previously described (Sachdeva et al., 2014). The proteomic screen revealed that ∼300 proteins were differentially regulated at least 2-fold between the miR-16-expressing and miR-16-deleted sarcoma cells. This included 170 upregulated and 121 downregulated proteins (supplementary material Table S3). We subsequently focused on proteins whose mRNA has a putative binding site for miR-16 using Targetscan and found that only 11 of the ∼170 upregulated proteins were encoded by genes containing predicted miR-16 binding sites (supplementary material Table S4). Some of these 11 genes have been implicated in metastasis, including *Carm1* (Wang et al., 2014), *Mylk* (Gurski et al., 2014), *Rac2* (Joshi et al., 2014), *Fra1* (Yang et al., 2013) and *Eno1* (Yu et al., 2012). Therefore, just as we recently demonstrated that miR-182 regulates metastasis by targeting multiple genes (Sachdeva et al., 2014), miR-16 may also regulate metastasis through a number of targets, which are differentially regulated in immunocompromised versus immunocompetent mice.

miR-16 was first reported to be frequently deleted and/or downregulated in chronic lymphocytic leukemia (CLL) at the 13q14.3 locus (Calin et al., 2002). Later, many investigators found downregulation of miR-16 in multiple tumor types, including prostate cancer, pituitary adenomas and gastric cancer (Bonci et al., 2008; Bottoni et al., 2005). A recent study found that miR-16 acts as a tumor suppressor in osteosarcoma (Jones et al., 2012). Using unbiased genomic profiling, miR-16 was found to be lost in human osteosarcoma specimens and was further shown to suppress tumor growth by activating caspase-3 in xenograft studies in nude mice. To our knowledge, our study reports for the first time that miR-16 is downregulated in both human and mouse metastatic STS. Although we did not observe any deletion of the miR-16 locus in primary metastatic mouse sarcomas by comparative genomic hybridization (data not shown), there could be other factors that regulate miR-16 expression within primary sarcomas. For example, expression of miR-16 is decreased by hypoxia (Dejean et al., 2011), and hypoxia also correlates with metastasis in STS (Brizel et al., 1996; Eisinger-Mathason et al., 2013). Therefore, one potential mechanism by which tumors might downregulate miR-16 to promote metastasis is via hypoxia.

In addition, we demonstrate that miR-16 can suppress *in vitro* migration and invasion of primary STS cells. Overexpression of miR-16 in sarcoma cells was sufficient to limit metastasis in an orthotopic assay in immunodeficient mice, which suggests that decreased miR-16 may be necessary for metastasis in some sarcomas. However, deletion of miR-16 failed to promote metastasis in a primary tumor model system, which suggests that loss of miR-16 is not sufficient to promote metastasis *in vivo*.

## MATERIALS AND METHODS

### Animals

All animal work was performed in accordance with Duke University Animal Care and Use Committee approved protocols. Primary soft tissue sarcomas were generated using the previously described alleles: *LSL-Kras^G12D^* (Jackson et al., 2001), *p53^Fl^* (Jonkers et al., 2001) and *miR-15a/16-1^Fl^* (Klein et al., 2010), in mice with a mixed genetic background. Tumors were generated by intramuscular injection of an adenovirus expressing Cre recombinase, as previously described (Kirsch et al., 2007) into the hind limb of mice with genotype *LSL-Kras^G12D/+^; p53^fl/fl^* (KP) or *LSL-Kras^G12D/+^; p53^fl/fl^; miR-15a/16-1^fl/fl^* (KP miR-16 F/F). Primary tumors were removed when they reached approximately 500 mm^3^ in volume. Mice were examined about 3 times per week for 200 days following amputations, until they developed signs of systemic illness, such as hunched posture, lethargy and ruffled fur. At this point, the mice were killed and the lungs analyzed for metastasis.

For the orthotopic metastasis assay, athymic nude (nu/nu) mice (5-6 weeks old) were purchased from Taconic labs (NCRNU-M) and were maintained in Duke University's accredited animal facility. Fifty thousand exponentially growing KP1 vector or KP1 miR-16 cells were injected into the hind limb muscle of the nude mice. When the tumor size reached about 250-350 mm^3^, the tumor-bearing limb was amputated and mice were followed for the development of lung metastases.

### Evaluation of metastases

For mice that developed lung metastases, at necropsy, macroscopic lung metastases were present. For all mice, lungs were fixed in formalin and 70% ethanol. After embedding the lungs in paraffin, sections were stained with hematoxylin and eosin for evaluation by a sarcoma pathologist (Diana M. Cardona, Department of Pathology, Duke University Medical Center, Durham, NC) blinded to the experimental variable, such as the genotype of the mice. Therefore, the presence or absence of lung metastases was based on macroscopic and microscopic evaluation of the lungs. To score the number of lung metastases and the area of lung with metastases per mouse, we imaged three independent areas of lung sections using a Leica DM5500B microscope with a 10× objective. The sections were quantified using Image Pro software (Media Cybernetics, Rockville, MD).

### Cell culture

Each cell line (KP1-KP3), was derived from a different primary sarcoma generated from individual KP mice. Sarcomas between 200 and 300 cm^3^ were harvested in cell culture hoods and cut into very small (approximately 0.25×0.25×0.25 cm) pieces. Then, they were digested using a solution containing 5 mg/ml collagenase, 2.4 U/ml dispase and 0.05% trypsin in phosphate buffer saline (PBS) for a minimum of 1 hour. The digested mixture/slurry was then filtered using a 70 μm filter followed by lysis of red blood cells using ammonium chloride/potassium buffer according to the manufacturer's protocol (Lonza). Lastly, cells were filtered using a 40 μm sieve and cultured for 5-8 passages to deplete stromal cells before they were used for the experiments. Cells were cultured in DMEM supplemented with 10% FBS and incubated at 37°C with 5% CO_2_ in a humidified cell culture incubator.

### Plasmids and transduction

The lentiviral vectors (System Biosciences) encoding miR-16 and other miRNAs were packaged and used to infect cell lines KP1-KP3, as described previously (Sachdeva et al., 2012). Briefly, pre-miR-16, pre-miR-146, pre-miR-223 and pre-miR-342 sequences were first PCR amplified using mouse genomic DNA as a template and Platinum *Taq* polymerase enzyme (Invitrogen) with corresponding specific primers. Primer sequences are shown in supplementary material Table S2. The amplified fragment was then cloned into a lentiviral vector (pCDH-CMV-MCS-EF1-copGFP from System Biosciences, Mountain View, CA) at *EcoR*I and *Not*I sites using the Choo-Choo cloning kit per the manufacturer's protocol (MCLAB, San Francisco, CA). The lentiviral vector was packaged using the pPACKH1 Lentivector Packaging Kit (Systems Biosciences, Mountain View, CA).

### Immunohistochemistry

Immunohistochemistry was performed using ready-to-use target retrieval solution (DAKO), antibody diluent (DAKO), the Vectastain Elite ABC Kit (Vector Labs, Burlingame, CA) and 3,3′-diaminobenzidine tetrahydrochloride (Sigma-Aldrich). To assess cell proliferation, we stained for Ki67 as previously described (Mito et al., 2013). Primary sarcomas were fixed in 10% formalin, 70% ethanol and paraffin embedded. Sections were cut at 5 μm and unstained sections were subjected to immunohistochemistry using an antibody against Ki67 (cat. no. 550609 from BD, San Jose, CA) diluted at 1:100. Three high-power (40×) fields were scored for Ki67 per mouse using ImageJ software and sarcomas from 10 mice per genotype were analyzed.

### Real-time RT-PCR

Human soft tissue sarcoma samples were acquired from MD Anderson under a protocol approved by the Duke University and MD Anderson Institutional Review Boards. The human sarcomas included in the study are undifferentiated pleomorphic sarcomas (UPS). Total RNA was extracted from 25 frozen tumor samples using Trizol, and cDNA synthesis was performed for 377 miRNAs using the Megamir TaqMan MicroRNAs Reverse Transcription Kit, followed by quantitative PCR with its respective probe, per the manufacturer's recommendation (Applied Biosystems, Foster City, CA). miRNA expression was calculated using the ΔΔCT method (Livak and Schmittgen, 2001) after normalization to SnoRNA234 expression. For mouse sarcomas, 64 tumors annotated for metastatic outcome were utilized (met, *N*=25; non-met, *N*=39). Samples were macro-dissected, stored in RNALater, homogenized in liquid nitrogen and total RNA was isolated using Trizol reagent (Invitrogen) per the manufacturer's recommendation. TaqMan arrays were performed for 335 miRNAs as described previously (Mito et al., 2013). Samples were compared using the ΔΔCT method by normalizing to SnoRNA202 expression and the median array. For both human and mouse, samples were clustered using sparse hierarchical clustering (Witten and Tibshirani, 2010) using average linkage. The heat map was made using differentially expressed miRNAs based on a two-tailed *t*-test (*P*<0.05) between primary tumors that did or did not give rise to distant metastases.

### Cell proliferation assay

Cell growth assays were carried out by MTT [3-(4,5-dimethylthiazol-2-yl)-2,5-diphenyltetrazolium bromide] assay, as previously described (Sachdeva et al., 2009). Briefly, cells were seeded in 96-well plates and incubated for the indicated number of days before application of MTT and colorimetric analysis.

### Migration and invasion assay

The 24-multiwell FluoroBlok Insert System, with a 24-well plate and lid and pore size of 8.0 µm (BD Biosciences, San Jose, CA) was used to determine the effect of overexpressed miRNAs on migration and invasiveness following the manufacturer's protocol. Infected cells were serum starved overnight, trypsinized the next day, resuspended in serum-free medium and then transferred to the hydrated Matrigel chambers (∼25,000 cells/well). The chambers were then incubated for 24 h in DMEM supplemented with 10% FBS in the bottom chambers before examination. The cells on the upper surface were scraped and washed away while the invaded cells on the lower surface were stained with Calcein AM dye for 1 h at 37°C. Finally, invaded cells were counted under a microscope, and the relative number was calculated. All the fluoroscence images were taken with 10× objective using a Zeiss Axiovert 25 microscope.

### Liquid chromatography/mass spectroscopy

Proteomic analysis was performed at the Duke Proteomics Core Facility (DPCF). Three cell pellets each from either miR-16 WT or miR-16 F/F cells were washed with 50 mM ammonium bicarbonate and solubilized by sonication in 200 µl of 0.2% Rapigest-SF (w/v). Protein concentration was then determined using a Bradford assay (Bio-Rad) and 30 µg of each sample was normalized to approximately 0.5 µg/µl in ammonium bicarbonate. Samples were reduced with 10 mM DTT at 80°C for 15 min and alkylated with iodoacetamide at room temperature in the dark for 30 min. Samples were then subjected to trypsin digestion overnight at 37°C at an enzyme to protein ratio (w/w) of 1:50. After digestion, all samples were acidified with 1% TFA and heated at 60°C for 2 h to hydrolyze Rapigest, centrifuged at 15,000 rpm for 5 min, and the supernatants were lyophilized. Samples were resuspended at 1 µg/µl in 200 mM ammonium formate (pH 10) liquid chromatography buffer containing 25 fmol/µl trypsinized yeast alcohol dehydrogenase 1 (MassPrep, Waters Corp., Milford, MA) as an internal standard. Quantitative two-dimensional liquid chromatography – tandem mass spectrometry (LC/LC-MS/MS) was performed on 3 µg of protein digest per sample in singlicate, and the pool was analyzed in triplicate with 3 µg injections (once each at the beginning, middle and end of the queue). The method uses two-dimensional liquid chromatography in a high-low pH reversed phase/reversed phase configuration on a nanoAcquity UPLC system (Waters) coupled to a Synapt G2 HDMS high resolution accurate mass tandem mass spectrometer (Waters) with nanoelectrospray ionization in a manner similar to previously described (Gilar et al., 2005a,b). The total analysis cycle time for each sample injection was approximately 6 h. Statistical significance was determined by a two-tailed Student's *t*-test on log_2_-transformed data.

### Statistical analyses

Statistical significance of the studies was analyzed by two-tailed Student's *t*-test, Mann–Whitney test, or one-way ANOVA. Differences with *P* values less than 0.05 were considered significant. The specific statistical test used for each analysis is indicated in the figure legends.

## Supplementary Material

Supplementary Material
