# MicroRNA-16 suppresses metastasis in an orthotopic, but not autochthonous, mouse model of soft tissue sarcoma

**DOI:** 10.1242/dmm.022582

**Published:** 2015-09-01

**Authors:** Mohit Sachdeva, Melodi J. Whitley, Jeffrey K. Mito, Yan Ma, Dina C. Lev, Diana M. Cardona, David G. Kirsch

There was an error published in *Dis. Model. Mech.* **8**, 867-875.

The spelling of an author name was given incorrectly as Melody J. Whitley. The correct spelling is given in the author list above.

The authors apologise to readers for this error.

