# Supplementary material for: MicroRNA-16 suppresses metastasis in an orthotopic, but not autochthonous, mouse model of soft tissue sarcoma
Source: Dis Model Mech. 2015 Aug 1;8(8):867–75. doi: 10.1242/dmm.017897 (PMC4527278; doi:10.1242/dmm.017897)
Supplement: Supplementary Material [file supp_8_8_867__index.html]

Supplementary Material 

# MicroRNA-16 suppresses metastasis in an orthotopic, but not autochthonous, mouse model of soft tissue sarcoma

## DMM017897 Supplementary Material

- Supplementary Material
