# Supplementary material for: MicroRNA-16 suppresses metastasis in an orthotopic, but not autochthonous, mouse model of soft tissue sarcoma
Source: Dis Model Mech. 2015 Aug 1;8(8):867–75. doi: 10.1242/dmm.017897 (PMC4527278; doi:10.1242/dmm.017897)
Supplement: Supplementary Material [file supp_017897_DMM017897supp.pdf]

Supplementary Tables

Table S1. List of differentially expressed miRNAs between non-metastatic versus metastatic primary human sarcomas using miRNA TLDA-array.

MicroRNA	Fold Change (Met/Non-met)	p-value
hsa-miR-518b	0.12	0.009
hsa-miR-511	0.13	0.003
hsa-miR-629	0.17	0.006
hsa-miR-150	0.19	0.015
hsa-miR-146a	0.2	0.005
hsa-miR-519a	0.2	0.008
hsa-miR-223	0.2	0.013
hsa-miR-107	0.23	0.020
hsa-miR-627	0.24	0.007
hsa-miR-125a-3p	0.24	0.023
hsa-miR-140-3p	0.26	0.005
hsa-miR-126	0.26	0.015
hsa-miR-545	0.29	0.012
hsa-miR-489	0.29	0.035
hsa-miR-142-3p	0.3	0.010
hsa-miR-16	0.3	0.016
hsa-miR-21	0.3	0.019
hsa-miR-222	0.33	0.016
hsa-miR-148b	0.33	0.028

hsa-miR-196b	0.33	0.045
hsa-miR-454	0.34	0.014
hsa-miR-185	0.34	0.030
hsa-miR-342-3p	0.35	0.013
hsa-miR-191	0.35	0.014
hsa-miR-423-5p	0.35	0.028
hsa-miR-190	0.35	0.043
hsa-miR-29a	0.36	0.004
hsa-miR-103	0.37	0.006
hsa-miR-339-3p	0.37	0.026
hsa-miR-320	0.37	0.034
hsa-miR-152	0.38	0.019
hsa-miR-197	0.39	0.002
hsa-miR-28-3p	0.39	0.013
hsa-miR-340	0.39	0.027
hsa-miR-186	0.4	0.011
hsa-miR-590-5p	0.41	0.023
hsa-miR-484	0.42	0.032
hsa-miR-125a-5p	0.44	0.011
hsa-miR-24	0.45	0.021
hsa-miR-374a	0.46	0.037
hsa-miR-425	0.47	0.047
hsa-miR-574-3p	0.48	0.023
hsa-miR-374b	0.48	0.049

Table S2. Primers used in the study.

Name	Direction	Sequence	Use
miR-16	Forward<br>Reverse	ctagagctagcgaattcctgtaaattactattgaggt<br>gcagatccttgcggccgcgatgttaagcctctaattct	Cloning
miR-223	Forward<br>Reverse	ctagagctagcgaattcgcagtccatggcattttcac<br>gcagatccttgcggccgcgaaggaacacataaattgtc	Cloning
miR-146a	Forward<br>Reverse	ctagagctagcgaattcgcttgcaccacatgcccagc<br>gcagatccttgcggccgccaagatattttatcagagca	Cloning
miR-342	Forward<br>Reverse	ctagagctagcgaattccatgttggactgaactgcttt<br>gcagatccttgcggccgccctaagagactgacatgcaa	Cloning

Table S3. List of differentially expressed proteins between miR-16 WT and miR-16 deleted primary mouse sarcoma cells from a LC/MS proteomic screen. Only proteins with a change of 2 fold or more are included in this list.

Upregulated proteins in miR-16 F/F versus miR-16 WT sarcoma cell lines (Greater than 2-fold change)

Protein Description	Peptides Count	P-value	Fold Change KO/WT
Adenylate kinase isoenzyme 1 OS=Mus musculus GN=Ak1 PE=1 SV=1	1	0.01	25.765
Switch-associated protein 70 OS=Mus musculus GN=Swap70 PE=1 SV=2	1	0.008	17.71
SH3 and multiple ankyrin repeat domains protein 3 OS=Mus musculus GN=Shank3 PE=1 SV=2	1	0.005	16.366
THAP domain-containing protein 2 OS=Mus musculus GN=Thap2 PE=2 SV=1	1	0.032	11.513
Ubiquitin carboxyl-terminal hydrolase isozyme L1 OS=Mus musculus GN=Uchl1 PE=1 SV=1	1	6.54E-12	11.087
Glycerol-3-phosphate acyltransferase 3 OS=Mus musculus GN=Agpat9 PE=1 SV=1	1	0.135	8.245
Isoform 2 of Protein piccolo OS=Mus musculus GN=Pclo	1	0.042	6.227
Cellular retinoic acid-binding protein 1 OS=Mus musculus GN=Crabp1 PE=1 SV=2	11	3.85E-15	6.118
Isoform 2 of THUMP domain-containing protein 3 OS=Mus musculus GN=Thumpd3	1	3.18E-06	5.759
Integrator complex subunit 3 OS=Mus musculus GN=Ints3 PE=1 SV=2	1	0.109	5.619
Nuclear ubiquitous casein and cyclin-dependent kinases substrate OS=Mus musculus GN=Nucks1 PE=1 SV=1	1	0.117	5.399
Malcavernin OS=Mus musculus GN=Ccm2 PE=1 SV=1	1	0.073	5.397
Gag polyprotein OS=Hortulanus murine leukemia virus GN=gag PE=3 SV=3	1	3.69E-15	5.075
Tyrosine-protein kinase transmembrane receptor ROR1 OS=Mus musculus GN=Ror1 PE=2 SV=1	1	0.021	5.041

4-hydroxyphenylpyruvate dioxygenase-like protein OS=Mus musculus GN=Hpdl PE=2 SV=1	1	0.005	4.809
Isoform 2 of A-kinase anchor protein SPHKAP OS=Mus musculus GN=Sphkap	1	4.95E-14	4.673
Mitotic spindle assembly checkpoint protein MAD2A OS=Mus musculus GN=Mad2l1 PE=2 SV=2	1	0.111	4.61
Isoform 2 of Zinc finger protein AEBP2 OS=Mus musculus GN=Aebp2	1	0.136	4.605
Isoform B of Heat shock protein beta-1 OS=Mus musculus GN=Hspb1	1	0.021	4.457
Uncharacterized protein C7orf50 homolog OS=Mus musculus PE=2 SV=2	1	0.041	4.339
Nuclear receptor-interacting protein 1 OS=Mus musculus GN=Nrip1 PE=1 SV=1	1	0.025	4.224
Isoform 1 of Dual specificity mitogen-activated protein kinase kinase 3 OS=Mus musculus GN=Map2k3	1	0.053	4.218
Isoform 2 of Rab11 family-interacting protein 3 OS=Mus musculus GN=Rab11fip3	1	0.151	4.215
Ras-related protein Rab-37 OS=Mus musculus GN=Rab37 PE=1 SV=2	2	0.046	4.189
Cellular retinoic acid-binding protein 2 OS=Mus musculus GN=Crabp2 PE=2 SV=2	3	9.94E-06	4.175
Retinol-binding protein 1 OS=Mus musculus GN=Rbp1 PE=2 SV=2	1	1.16E-08	4.143
Isoform 1 of Ninein-like protein OS=Mus musculus GN=Ninl	1	0.045	4.127
Glutamate--cysteine ligase catalytic subunit OS=Mus musculus GN=Gclc PE=2 SV=4	1	0.112	3.917
InaD-like protein OS=Mus musculus GN=Inadl PE=1 SV=2	1	0.036	3.889
CDK5 regulatory subunit-associated protein 2 OS=Mus musculus GN=Cdk5rap2 PE=1 SV=3	1	0.069	3.807
Chondroitin sulfate N-acetylgalactosaminyltransferase 1 OS=Mus musculus GN=Csgalnact1 PE=2 SV=1	2	0.109	3.793
Uncharacterized protein C3orf23 homolog OS=Mus musculus GN=D9Ertd402e PE=2 SV=1	1	0.015	3.779
Methionine synthase OS=Mus musculus GN=Mtr PE=2 SV=1	1	0.036	3.752
Methylcytosine dioxygenase TET2 OS=Mus musculus GN=Tet2 PE=1 SV=3	1	1.77E-04	3.737

T-cell-specific surface glycoprotein CD28 OS=Mus musculus GN=Cd28 PE=2 SV=1	1	3.14E-08	3.71
Isoform 2 of Tenascin OS=Mus musculus GN=Tnc	1	0.127	3.584
EH domain-containing protein 1 OS=Mus musculus GN=Ehd1 PE=1 SV=1	1	6.28E-05	3.469
Eukaryotic translation initiation factor 2D OS=Mus musculus GN=Eif2d PE=2 SV=3	1	0.108	3.366
Osteopontin OS=Mus musculus GN=Spp1 PE=1 SV=1	2	0.054	3.316
Sorting nexin-5 OS=Mus musculus GN=Snx5 PE=1 SV=1	1	0.071	3.285
Isoform 2 of Protein KIAA1731 homolog OS=Mus musculus GN=Kiaa1731	1	0.008	3.222
Poly [ADP-ribose] polymerase 12 OS=Mus musculus GN=Parp12 PE=2 SV=2	1	0.012	3.188
Oxysterol-binding protein-related protein 3 OS=Mus musculus GN=Osbpl3 PE=1 SV=1	3	0.047	3.17
Ankycorbin OS=Mus musculus GN=Rai14 PE=1 SV=1	1	0.064	3.159
Acetoacetyl-CoA synthetase OS=Mus musculus GN=Aacs PE=1 SV=1	2	2.11E-15	3.133
ADP-ribosylation factor-like protein 3 OS=Mus musculus GN=Arl3 PE=1 SV=1	1	0.119	3.068
Testin OS=Mus musculus GN=Tes PE=2 SV=1	1	0.017	3.042
Dihydropyrimidinase-related protein 3 OS=Mus musculus GN=Dpysl3 PE=1 SV=1	15	0	3.041
Dihydropteridine reductase OS=Mus musculus GN=Qdpr PE=1 SV=2	3	0.002	3.034
Oxidized low-density lipoprotein receptor 1 OS=Mus musculus GN=Olr1 PE=2 SV=1	1	0.052	3.015
Peptidyl-prolyl cis-trans isomerase-like 2 OS=Mus musculus GN=Ppil2 PE=2 SV=2	1	0.067	3.012
Probable glutathione peroxidase 8 OS=Mus musculus GN=Gpx8 PE=2 SV=1	3	4.37E-27	3.002
Ubiquitin carboxyl-terminal hydrolase 47 OS=Mus musculus GN=Usp47 PE=1 SV=2	1	1.45E-07	2.997
DNA2-like helicase OS=Mus musculus GN=Dna2 PE=2 SV=2	1	0.009	2.973
Serine/threonine-protein kinase PLK1 OS=Mus musculus GN=Plk1 PE=1 SV=2	1	0.122	2.926
Oxysterol-binding protein 1 OS=Mus musculus GN=Osbp PE=1 SV=2	1	1.45E-05	2.92
Spermatogenesis-associated protein 5 OS=Mus musculus GN=Spata5 PE=2	2	2.70E-05	2.889

SV=2			
Myosin light chain kinase, smooth muscle OS=Mus musculus GN=Mylk PE=1 SV=3	1	0.059	2.88
Methyl-CpG-binding domain protein 2 OS=Mus musculus GN=Mbd2 PE=2 SV=1	1	0.007	2.838
Myosin light chain 4 OS=Mus musculus GN=Myl4 PE=2 SV=3	3	0.038	2.826
Inner centromere protein OS=Mus musculus GN=Incenp PE=1 SV=2	1	0.013	2.81
Keratin, type II cytoskeletal 2 oral OS=Mus musculus GN=Krt76 PE=2 SV=1	1	0.045	2.802
Protein HIRA OS=Mus musculus GN=Hira PE=1 SV=3	1	2.90E-27	2.784
Isoform 2 of E3 UFM1-protein ligase 1 OS=Mus musculus GN=Ufl1	1	0.134	2.778
Coiled-coil and C2 domain-containing protein 1B OS=Mus musculus GN=Cc2d1b PE=1 SV=1	1	2.22E-19	2.768
Isoform 2 of Membrane metallo-endopeptidase-like 1 OS=Mus musculus GN=Mmel1	1	0.137	2.758
Poly(rC)-binding protein 3 OS=Mus musculus GN=Pcbp3 PE=2 SV=3	1	9.29E-06	2.729
Parathymosin OS=Mus musculus GN=Ptms PE=2 SV=3	1	0.032	2.722
Striatin OS=Mus musculus GN=Strn PE=1 SV=1	1	3.51E-06	2.703
PDZ and LIM domain protein 1 OS=Mus musculus GN=Pdlim1 PE=2 SV=4	7	9.49E-09	2.699
Isoform 2 of SET domain-containing protein 3 OS=Mus musculus GN=Setd3	1	0.028	2.691
Isoform 2 of LisH domain-containing protein ARMC9 OS=Mus musculus GN=Armc9	1	0.169	2.678
Isoform 2 of Histone-lysine N-methyltransferase SUV39H1 OS=Mus musculus GN=Suv39h1	1	0.254	2.656
1-phosphatidylinositol-4,5-bisphosphate phosphodiesterase epsilon-1 OS=Mus musculus GN=Plce1 PE=1 SV=2	1	0.057	2.63
Importin-7 OS=Mus musculus GN=Ipo7 PE=1 SV=2	3	1.32E-16	2.62
Isoform 2 of Coiled-coil domain-containing protein 50 OS=Mus musculus GN=Ccdc50	1	0.01	2.619
Ectonucleoside triphosphate diphosphohydrolase 2 OS=Mus musculus GN=Entpd2 PE=1 SV=2	1	0.068	2.614
Craniofacial development protein 1 OS=Mus musculus GN=Cfdp1 PE=1 SV=1	1	2.20E-04	2.588

Hypoxanthine-guanine phosphoribosyltransferase OS=Mus musculus GN=Hprt1 PE=1 SV=3	3	4.07E-11	2.587
Isoform 2 of Pre-rRNA-processing protein TSR1 homolog OS=Mus musculus GN=Tsr1	1	0.001	2.584
La-related protein 7 OS=Mus musculus GN=Larp7 PE=1 SV=2	2	0.009	2.563
Uncharacterized protein C2orf63 homolog OS=Mus musculus PE=2 SV=1	1	0.097	2.563
H-2 class I histocompatibility antigen, Q10 alpha chain OS=Mus musculus GN=H2-Q10 PE=1 SV=3	1	0.03	2.558
Leucine-rich repeat flightless-interacting protein 1 OS=Mus musculus GN=Lrrfip1 PE=1 SV=2	2	1.57E-05	2.53
Cytosolic non-specific dipeptidase OS=Mus musculus GN=Cndp2 PE=1 SV=1	2	3.20E-09	2.527
Glutamate--cysteine ligase regulatory subunit OS=Mus musculus GN=Gclm PE=2 SV=1	2	2.51E-04	2.523
Glucosylceramidase OS=Mus musculus GN=Gba PE=1 SV=1	3	0.034	2.514
Collagen triple helix repeat-containing protein 1 OS=Mus musculus GN=Cthrc1 PE=2 SV=1	3	0.066	2.509
Fos-related antigen 1 OS=Mus musculus GN=Fosl1 PE=2 SV=2	1	0.008	2.506
Isoform 2 of Glutathione S-transferase C-terminal domain-containing protein OS=Mus musculus GN=Gstcd	1	0.09	2.494
Isoform 2 of Mannan-binding lectin serine protease 1 OS=Mus musculus GN=Masp1	1	0.236	2.477
Platelet-activating factor acetylhydrolase IB subunit gamma OS=Mus musculus GN=Pafah1b3 PE=1 SV=1	1	9.11E-05	2.475
Arachidonate 5-lipoxygenase OS=Mus musculus GN=Alox5 PE=1 SV=2	2	0.071	2.474
2-amino-3-ketobutyrate coenzyme A ligase, mitochondrial OS=Mus musculus GN=Gcat PE=1 SV=2	3	0.011	2.459
Matrilin-2 OS=Mus musculus GN=Matn2 PE=2 SV=1	1	0.08	2.448
Alpha-1,6-mannosyl-glycoprotein 2-beta-N-acetylglucosaminyltransferase OS=Mus musculus GN=Mgat2 PE=2 SV=1	2	6.24E-22	2.421
Eukaryotic translation initiation factor 4 gamma 2 OS=Mus musculus GN=Eif4g2 PE=1 SV=2	1	3.77E-07	2.405

Protein FAM111A OS=Mus musculus GN=Fam111a PE=2 SV=1	1	2.42E-06	2.387
Thyroid receptor-interacting protein 13 OS=Mus musculus GN=Trip13 PE=2 SV=1	1	1.92E-08	2.385
Three prime repair exonuclease 1 OS=Mus musculus GN=Trex1 PE=1 SV=2	1	0.003	2.385
ADP-ribosylation factor 1 OS=Mus musculus GN=Arf1 PE=1 SV=2	2	0.012	2.381
Aquaporin-1 OS=Mus musculus GN=Aqp1 PE=1 SV=3	2	5.70E-10	2.377
Transcription factor AP-1 OS=Mus musculus GN=Jun PE=1 SV=3	2	1.65E-09	2.363
Isoform 2 of Serine/threonine-protein phosphatase 2B catalytic subunit alpha isoform OS=Mus musculus GN=Ppp3ca	1	1.92E-08	2.358
60S ribosomal protein L7-like 1 OS=Mus musculus GN=Rpl7l1 PE=2 SV=1	4	0.182	2.351
Inositol 1,4,5-trisphosphate receptor type 3 OS=Mus musculus GN=Itpr3 PE=1 SV=3	6	0.047	2.346
NudC domain-containing protein 2 OS=Mus musculus GN=Nudcd2 PE=1 SV=1	1	5.75E-12	2.345
Golgin subfamily A member 4 OS=Mus musculus GN=Golga4 PE=1 SV=2	1	0.099	2.341
Fanconi anemia-associated protein of 24 kDa OS=Mus musculus GN=Faap24 PE=2 SV=1	2	0.002	2.339
Transcription elongation factor A N-terminal and central domain-containing protein OS=Mus musculus GN=Tceanc PE=2 SV=1	1	0.009	2.325
Hematopoietic progenitor cell antigen CD34 OS=Mus musculus GN=Cd34 PE=1 SV=1	2	6.32E-07	2.324
Isoform 2 of Tetratricopeptide repeat protein 14 OS=Mus musculus GN=Ttc14	1	0.097	2.321
Transcription elongation factor B polypeptide 1 OS=Mus musculus GN=Tceb1 PE=1 SV=1	1	0.053	2.311
Isoform 4 of General vesicular transport factor p115 OS=Mus musculus GN=Uso1	1	1.05E-06	2.29
UPF0485 protein C1orf144 homolog OS=Mus musculus GN=D4Ertd22e PE=1 SV=1	1	4.34E-07	2.254
Adenomatous polyposis coli protein 2 OS=Mus musculus GN=Apc2 PE=1 SV=1	1	0.037	2.25
Citrate lyase subunit beta-like protein, mitochondrial OS=Mus musculus	1	0.008	2.241

GN=Clybl PE=2 SV=2			
Protein FRG1 OS=Mus musculus GN=Frg1 PE=1 SV=2	1	0.054	2.235
Allograft inflammatory factor 1-like OS=Mus musculus GN=Aif1l PE=2 SV=1	2	4.25E-05	2.216
S-formylglutathione hydrolase OS=Mus musculus GN=Esd PE=2 SV=1	19	7.71E-05	2.212
Cadherin EGF LAG seven-pass G-type receptor 3 OS=Mus musculus GN=Celsr3 PE=2 SV=1	1	0.113	2.204
Son of sevenless homolog 1 OS=Mus musculus GN=Sos1 PE=1 SV=2	1	0.281	2.195
1,4-alpha-glucan-branching enzyme OS=Mus musculus GN=Gbe1 PE=2 SV=1	1	0.004	2.186
DnaJ homolog subfamily B member 1 OS=Mus musculus GN=Dnajb1 PE=2 SV=3	1	1.00E-05	2.184
Replication factor C subunit 2 OS=Mus musculus GN=Rfc2 PE=2 SV=1	1	0.282	2.181
ATP-binding cassette sub-family D member 4 OS=Mus musculus GN=Abcd4 PE=2 SV=1	1	0.195	2.179
6-phosphogluconolactonase OS=Mus musculus GN=Pgls PE=2 SV=1	6	2.07E-08	2.173
Sarcoplasmic/endoplasmic reticulum calcium ATPase 1 OS=Mus musculus GN=Atp2a1 PE=2 SV=1	1	0.089	2.161
L-lactate dehydrogenase A chain OS=Mus musculus GN=Ldha PE=1 SV=3	28	2.59E-06	2.15
Heme oxygenase 1 OS=Mus musculus GN=Hmox1 PE=1 SV=1	1	0.033	2.149
Ras-related protein Rab-26 OS=Mus musculus GN=Rab26 PE=2 SV=1	1	0.101	2.149
Ufm1-specific protease 2 OS=Mus musculus GN=Ufsp2 PE=1 SV=1	1	1.58E-09	2.138
Four and a half LIM domains protein 3 OS=Mus musculus GN=Fhl3 PE=2 SV=1	2	0.004	2.138
Alpha-enolase OS=Mus musculus GN=Eno1 PE=1 SV=3	44	1.69E-10	2.136
Acidic leucine-rich nuclear phosphoprotein 32 family member B OS=Mus musculus GN=Anp32b PE=1 SV=1	8	0.008	2.126
Isoform 2 of Probable cation-transporting ATPase 13A4 OS=Mus musculus GN=Atp13a4	1	0.142	2.126
R3H domain-containing protein C19orf22 homolog OS=Mus musculus PE=2 SV=1	1	0.012	2.119
Actin-related protein 2 OS=Mus musculus GN=Actr2 PE=1 SV=1	3	3.20E-06	2.117
Eukaryotic translation initiation factor 3	1	0.025	2.112

subunit E OS=Mus musculus GN=Eif3e PE=1 SV=1			
Low molecular weight phosphotyrosine protein phosphatase OS=Mus musculus GN=Acp1 PE=2 SV=3	5	1.51E-10	2.111
S-methyl-5'-thioadenosine phosphorylase OS=Mus musculus GN=Mtap PE=2 SV=1	6	5.60E-04	2.105
Proliferating cell nuclear antigen OS=Mus musculus GN=Pcna PE=1 SV=2	11	5.10E-05	2.103
Histone-arginine methyltransferase CARM1 OS=Mus musculus GN=Carm1 PE=1 SV=2	1	1.22E-07	2.102
28S ribosomal protein S33, mitochondrial OS=Mus musculus GN=Mrps33 PE=2 SV=1	1	0.321	2.092
Cell growth-regulating nucleolar protein OS=Mus musculus GN=Lyar PE=1 SV=2	1	2.85E-05	2.088
Protein transport protein Sec31A OS=Mus musculus GN=Sec31a PE=1 SV=2	2	2.66E-11	2.086
Isoform 2 of Traf2 and NCK-interacting protein kinase OS=Mus musculus GN=Tnik	1	1.83E-04	2.067
Spermidine synthase OS=Mus musculus GN=Srm PE=2 SV=1	4	1.46E-18	2.066
CTP synthase 1 OS=Mus musculus GN=Ctps PE=1 SV=2	5	3.07E-10	2.058
Phosphoglycerate mutase 1 OS=Mus musculus GN=Pgam1 PE=1 SV=3	17	7.31E-12	2.051
Eukaryotic translation initiation factor 3 subunit J OS=Mus musculus GN=Eif3j PE=2 SV=1	2	5.84E-06	2.037
Isoform 2 of Poly [ADP-ribose] polymerase 9 OS=Mus musculus GN=Parp9	1	1.74E-04	2.032
Importin-9 OS=Mus musculus GN=Ipo9 PE=1 SV=3	2	0.071	2.031
Ras-related protein Rab-8B OS=Mus musculus GN=Rab8b PE=1 SV=1	2	0.058	2.029
Hydroxyacid oxidase 2 OS=Mus musculus GN=Hao2 PE=2 SV=1	1	1.00E-05	2.026
Cerebellin-4 OS=Mus musculus GN=Cbln4 PE=1 SV=1	1	0.004	2.025
Isoform A1 of Microphthalmia-associated transcription factor OS=Mus musculus GN=Mitf	1	1.05E-04	2.024
Ras-related C3 botulinum toxin substrate 2 OS=Mus musculus GN=Rac2 PE=2 SV=1	1	0.142	2.024
Ephrin type-A receptor 4 OS=Mus musculus GN=Epha4 PE=1 SV=1	1	7.57E-04	2.021
Coiled-coil-helix-coiled-coil-helix domain-	1	0.176	2.019

containing protein 1 OS=Mus musculus GN=Chchd1 PE=2 SV=1			
Isoform 3 of Submandibular gland protein C OS=Mus musculus GN=Muc19	1	0.157	2.014
Transgelin OS=Mus musculus GN=Tagln PE=1 SV=3	1	0.078	2.011
Abhydrolase domain-containing protein 2 OS=Mus musculus GN=Abhd2 PE=2 SV=1	1	0.203	2.009
AP-1 complex subunit gamma-1 OS=Mus musculus GN=Ap1g1 PE=1 SV=3	2	2.18E-16	2.002
Twinfilin-2 OS=Mus musculus GN=Twf2 PE=1 SV=1	3	4.75E-07	1.997
Ubiquitin carboxyl-terminal hydrolase isozyme L3 OS=Mus musculus GN=Uchl3 PE=1 SV=2	1	4.47E-06	1.997
Calcium-regulated heat stable protein 1 OS=Mus musculus GN=Carhsp1 PE=1 SV=1	2	1.30E-07	1.995
Uridine phosphorylase 1 OS=Mus musculus GN=Upp1 PE=1 SV=2	2	0.002	1.995
Acyl-CoA synthetase family member 3, mitochondrial OS=Mus musculus GN=Acsf3 PE=2 SV=2	2	0.004	1.993

Table S4. Genes upregulated in the proteomic screen that have putative miR-16 binding sites (N=11).

Upregulated genes in the proteomic screen with a miR-16 binding motif	Fold upregulated	Number of putative miR-16 binding sites	Binding site conserved (C)/only in mouse (NC)
HIRA	2.78	1	C
AACS	3.1	1	NC
CD28	3.7	2	C
CARM1	2.1	1	C
MINK1	2.06	1	C
FOSL1	2.5	1	C
SETD3	2.69	1	C
MYLK	2.88	1	C
RAC2	2	1	NC
ENO1	2.2	1	NC
AK1	25	1	NC
